# Ketamine Alters Functional Gamma and Theta Resting-State Connectivity in Healthy Humans: Implications for Schizophrenia Treatment Targeting the Glutamate System

**DOI:** 10.3389/fpsyt.2021.671007

**Published:** 2021-06-10

**Authors:** Stjepan Curic, Christina Andreou, Guido Nolte, Saskia Steinmann, Stephanie Thiebes, Nenad Polomac, Moritz Haaf, Jonas Rauh, Gregor Leicht, Christoph Mulert

**Affiliations:** ^1^Psychiatry Neuroimaging Branch, Department of Psychiatry and Psychotherapy, University Medical Center Hamburg-Eppendorf, Hamburg, Germany; ^2^Institute for Sex Research, Sexual Medicine and Forensic Psychiatry, Center of Psychosocial Medicine, University Medical Center Hamburg-Eppendorf, Hamburg, Germany; ^3^Translational Psychiatry Unit, Department of Psychiatry and Psychotherapy, University of Lübeck, Lübeck, Germany; ^4^Department of Neurophysiology and Pathophysiology, University Medical Center Hamburg-Eppendorf, Hamburg, Germany; ^5^Centre for Psychiatry and Psychotherapy, Justus Liebig University, Giessen, Germany

**Keywords:** resting state, gamma-band oscillations, theta-band oscillations, functional connectivity, ketamine model of schizophrenia, glutamate hypothesis, negative-symptoms

## Abstract

Disturbed functional connectivity is assumed to cause neurocognitive deficits in patients suffering from schizophrenia. A Glutamate N-methyl-D-aspartate receptor (NMDAR) dysfunction has been suggested as a possible mechanism underlying altered connectivity in schizophrenia, especially in the gamma- and theta-frequency range. The present study aimed to investigate the effects of the NMDAR-antagonist ketamine on resting-state power, functional connectivity, and schizophrenia-like psychopathological changes in healthy volunteers. In a placebo-controlled crossover design, 25 healthy subjects were recorded using resting-state 64-channel-electroencephalography (EEG) (eyes closed). The imaginary coherence-based *Multivariate Interaction Measure* (MIM) was used to measure gamma and theta connectivity across 80 cortical regions. The network-based statistic was applied to identify involved networks under ketamine. Psychopathology was assessed with the Positive and Negative Syndrome Scale (PANSS) and the 5-Dimensional Altered States of Consciousness Rating Scale (5D-ASC). Ketamine caused an increase in all PANSS (*p* < 0.001) as well as 5D-ASC scores (*p* < 0.01). Significant increases in resting-state gamma and theta power were observed under ketamine compared to placebo (*p* < 0.05). The source-space analysis revealed two distinct networks with an increased mean functional gamma- or theta-band connectivity during the ketamine session. The gamma-network consisted of midline regions, the cuneus, the precuneus, and the bilateral posterior cingulate cortices, while the theta-band network involved the Heschl gyrus, midline regions, the insula, and the middle cingulate cortex. The current source density (CSD) within the gamma-band correlated negatively with the PANSS negative symptom score, and the activity within the gamma-band network correlated negatively with the subjective *changed meaning of percepts* subscale of the 5D-ASC. These results are in line with resting-state patterns seen in people who have schizophrenia and argue for a crucial role of the glutamate system in mediating dysfunctional gamma- and theta-band-connectivity in schizophrenia. Resting-state networks could serve as biomarkers for the response to glutamatergic drugs or drug development efforts within the glutamate system.

## Introduction

Synchronized neural oscillations are essential for cognition, perception, and consciousness (1). Both oscillations in the low (theta) and high (gamma) frequency range coordinate communication between brain regions (2). While gamma-band oscillations (GBO) are crucial for both local and large-scale neuronal synchronization (3), theta-band-oscillations (TBO) enable long-range synchronization (4). It is widely believed that a disturbed formation of functioning networks is responsible for the characteristic symptomatology of schizophrenia (5). While task-specific oscillation patterns both in the theta- (6) and gamma-band (7) have been shown to be pathological among patients with schizophrenia, there is a growing body of evidence highlighting the significance of spontaneous, “resting-state” oscillatory activity relevant for schizophrenia (8–10). EEG and MEG studies have observed deviations in resting-state (RS) -GBO and -TBO patterns across different stages of schizophrenia: increased RS-GBO among patients with schizophrenia (11, 12); increased RS-GBO in the first episode of schizophrenia (13); increased RS-TBO in chronic schizophrenia and patients at high risk (14–17). Even patients with schizophrenia-like epilepsy showed increased RS-TBO compared to nonpsychotic epilepsy patients (18). Measures of connectivity that represent oscillatory coupling seem to be altered in people suffering from schizophrenia [for a review of the disconnection hypothesis of schizophrenia see (19)]: Patients with schizophrenia show increased RS-gamma- (20) and RS-theta-networks (15, 21, 22).

Gamma and theta oscillogenesis involves the interaction of glutamatergic pyramidal cells and parvalbumin- (PV^+^) and somatostatin-expressing (SST^+^) gamma-aminobutyric acid (GABA-) releasing -interneurons (23–25). The glutamate N-methyl-D-aspartate receptors (NMDAR) on these interneurons are essential for the synchronized inhibition provided by the GABAergic interneurons to generate both GBO and TBO (26–28). These findings are supported by post-mortem studies that found a reduced number of PV^+^-interneurons in the frontal cortex of patients with schizophrenia (29) and a reduced number of SST^+^-interneurons in various cortical tissues (DLPFC, PFC, hippocampus) of patients with schizophrenia (30).

Experiments with ketamine, a glutamate N-methyl-D-aspartate receptor (NMDAR)-antagonist, helped to identify the critical role of the neurotransmitter glutamate in the pathogenesis of schizophrenia (31): When given to healthy subjects, ketamine induces schizophrenia-like positive, negative, and cognitive symptoms (32, 33) and neurophysiological changes resembling schizophrenia [reviewed in Haaf et al. (34)]. Translational scientific approaches in NMDAR hypofunction animal models of schizophrenia [reviewed in Lee and Zhou (35)] and in healthy humans receiving ketamine (34, 36) parallel findings of patients with schizophrenia both in respect to psychopathological and neurophysiological changes. The need for novel glutamatergic treatment options for schizophrenia was reinforced by these findings and the unsatisfactory effect of conventional D2-targeting antipsychotics on the symptomatology of schizophrenia, especially on negative symptoms (37). While some studies showed promising results, suboptimal outcomes in other studies called for more insight into biomarkers to optimize and individualize glutamatergic treatments (38, 39).

The resting-state activity is a potential biomarker that can help predict or monitor the response of negative symptoms to glutamatergic substances (40). Still, only a few studies have investigated the role of RS oscillations, functional connectivity, networks, and the association with clinical symptoms in the ketamine model of schizophrenia (41–43).

While animal studies influencing the NMDA-R are consistent with NMDA-R-modulation in humans with respect to GBO, paralleling these findings with schizophrenia patients remains challenging, as both increased and decreased RS-GBOs have been observed in patients [reviewed by Bianciardi and Uhlhaas (44)]. This emphasizes the need for further studies characterizing the spectral characteristics in both the ketamine-model of schizophrenia and patient populations.

Founded on the strong association between glutamatergic neurotransmission, the PV^+^- and SST^+^-interneurons' role in gamma- and theta-oscillogenesis, the NMDAR functioning, and the need for glutamatergic biomarkers, this study aims to establish a translational link between evidence from preclinical studies and findings from studies investigating patients with schizophrenia. We hypothesized (1) a ketamine-induced schizophrenia-like increase in RS-GBO and RS-TBO, and (2) increased connectivity in the gamma and theta frequency range, along with (3) the manifestation of schizophrenia-like symptoms, as well as (4) a specific association between the ketamine-induced resting-state alterations and the severity of ketamine-induced symptoms.

## Materials and Methods

### Participants

The Ethics Committee of the Medical Association Hamburg approved the study. Participants were recruited via notice board at the University Medical Center Hamburg-Eppendorf (UKE) and word of mouth and consisted mainly of medical students and employees. Twenty-eight healthy subjects were enrolled and gave written informed consent according to the latest version of the Declaration of Helsinki. Two participants discontinued participation shortly before the first session; one of the participants dropped out due to adverse effects (dissociation/headache/nausea). For the following data analysis, twenty-five subjects with a mean age of 25 years (SD = 2.6) were included (for sociodemographic characteristics at baseline please see Supplementary Table 3).

This investigation was part of a larger study investigating ketamine-related, task- and stimulus-related neurophysiological measures in the same sample (45–47).

Exclusion criteria were acute or past psychiatric illness, tested with the Mini International Neuropsychiatric Interview (48) and the Schizotypal Personality Questionnaire (SPQ) (49), and health conditions that represented a contraindication to the administration of ketamine, assessed through a semi-structured interview. Participants with close relatives who have schizophrenia were excluded from the study.

### Psychometric Assessment

Before the first recording (baseline) and after both sessions (placebo/ketamine), an experienced psychiatrist conducted a psychopathological evaluation. The psychiatric symptomatology was assessed using the Positive and Negative Syndrome Scale (PANSS) (50) and the Altered State of Consciousness (5D-ASC) questionnaire (51). PANSS scores were evaluated using the five-factor model by van der Gaag et al. (52). The Altered State of Consciousness (5D-ASC) questionnaire assesses the subjective drug-effects and consists of 94 items representing key dimensions of altered states of consciousness. For this evaluation, the 11 subscales version, with improved homogeneity, compared to the original scale, was used (53).

### Study Design

In a randomized, placebo-controlled crossover study design, a subanesthetic dose of S-ketamine hydrochloride (Ketanest® S–Pfizer) was administered by an Infusomat® perfusor syringe pump (B. Braun, Melsungen, Germany) in a 0.9% sodium chloride (NaCl) solution for a total length of up to 75 min. An initial bolus of 10 mg of S-Ketamine was administered over 5 min, followed by a maintenance infusion of 0.006 mg/kg/min. As ketamine plasma levels accumulate with continuous infusion (54), the infusion rate was reduced by 10% every 10 min. The same procedure was applied during the placebo (0.9% NaCl) condition. The unblinded anesthesiologist did not inform the subjects or rating psychiatrist about the condition; however, ketamine-effects may have led to unblinding. The resting-state EEG was measured at the beginning of the session. An anesthesiologist monitored heart rate, blood pressure, oxygen saturation, and vigilance during both sessions.

### Resting State EEG Recording and Pre-processing

The EEG recording chamber was electrically shielded and sound-attenuated. The subjects were seated comfortably with their eyes closed while continuous EEG activity was recorded over 5–6 min. A 64-channels-EEG was recorded at a sampling rate of 1,000 Hz, with active electrodes mounted on an elastic cap (ActiCaps, Brain Products, Gilching, Germany) via the Brain Vision Recorder Software Version 1.10 (Brain Products, Gilching, Germany). Electrodes were positioned in an extended 10–20 system with the additional positions: AF7, AF3, AF4, AF8, F5, F1, F2, F6, F10, FT9, FT7, FC3, FC4, FT8, FT10, C5, C1, C2, C6, TP7, CPz, TP8, P5, P1, P2, P6, PO3, POz, and PO4. Eye movements were recorded by four EOG channels. Impedances were kept below 5 kΩ.

Offline preprocessing was carried out using the Brain Vision Analyzer (BVA) Version 2.1.0.327 (Brain Products, Gilching, Germany). After a Butterworth zero-phase band-pass filtering (0.1–100 Hz; 12 dB/octave), ocular and muscle artifacts were removed using an independent component analysis (ICA). The continuous EEG was segmented into epochs of 2,000 ms after re-referencing to the common average reference. A notch filter was applied to remove 50 Hz line noise.

For the scalp surface analysis, both the power and the current source density (CSD) were measured. The CSD was computed using the 4th order spherical spline interpolation and a maximal Degree of Legendre Polynomials of 10 (55). The EEG spectral analysis was based on a full-spectrum voltage Fast Fourier Transform (FFT) algorithm with a resolution of 0.5 Hz, which was used on the averaged data. The electrodes were grouped into different regions of laterality (left, right, middle) and region (frontal, temporo-central and posterior regions) for the pooled analysis. For precise electrode locations selected for electrode pools, please view the Supplementary Table 2. Mean values of the frequency-ranges (4–8 and 30–40 Hz) were computed for the scalp-level analysis. The spectra for both conditions were calculated with a grand average and a linear derivation.

Source space analyses were calculated in Matlab Version R2015a (Mathworks, Natick, USA) using custom-made scripts. Source-space localization analyses were computed using the exact low-resolution electromagnetic tomography (eLORETA) software (56). The center frequencies of the wavelet were set to 6 for the theta and 40 Hz for the gamma frequency range. Time-series were calculated for 80 source-space regions corresponding to the Automated Anatomical Labeling Atlas (40 for each hemisphere, see Supplementary Table 1 for the precise coordinates). Connectivity analyses were based on (80*79/2=) 3,160 pairs of sources. Zero-lag interactions from connectivity analyses were excluded, based on the concept that volume conduction occurs instantaneously, while true interactions occur at variable time delays (57). We used the *Multivariate Interaction Measure (MIM)* (58) for the connectivity analysis. This multivariate measure does not assume a fixed source direction at a given grid point and takes into account that the estimated activity at a given grid point reflects not only neural activation of that point but also of its vicinity.

### Statistical Analyses

Statistical analyses were performed using IBM SPSS Statistics Version 27. 5D-ASC scores were compared by paired-sample *t*-tests. PANSS scores were analyzed by repeated measure analyses of variance (RM-ANOVA) with the session (baseline, placebo, and ketamine) as within-subject factors. Scalp-level-differences in power and CSD were assessed by RM-ANOVA for each frequency band separately, with condition (placebo, ketamine), region (anterior, temporal-central, posterior), and laterality (left, middle, right) as factors. If Mauchly's test of sphericity indicated that the assumption of sphericity was violated, the degrees of freedom were corrected using the Greenhouse-Geisser estimates of sphericity. A multivariate linear regression analysis with the differences between the placebo and ketamine resting-state scalp- or network-activity as dependent variables and the differences between the intensity of schizophrenia-like symptoms as predictors (5 PANSS factors or 11 5D-ASC factors) were used to assess associations. *Post-hoc* bivariate Pearson's correlation coefficients explored the relationship between psychopathological changes and neurophysiological measures. Bonferroni corrections were applied to adjust for multiple comparisons.

For the source-level analysis, we used the network-based statistic (NBS), which corresponds to an application of cluster-based thresholding of statistical parametric maps to the graph model (59, 60). The open-source toolbox NBS Connectome v1.2 (http://www.nitrc.org/projects/nbs, last accessed on Feb 19, 2021) was used for the analysis (please see Supplementary Material for details). When comparing networks, the significance level was set to 0.025 because effects were tested in both directions. In all other analyses, the significance level was set to α = 0.05. The strength of the significant network was calculated for each subject and both conditions.

## Results

### Psychopathological Symptoms

**Figure 1:**
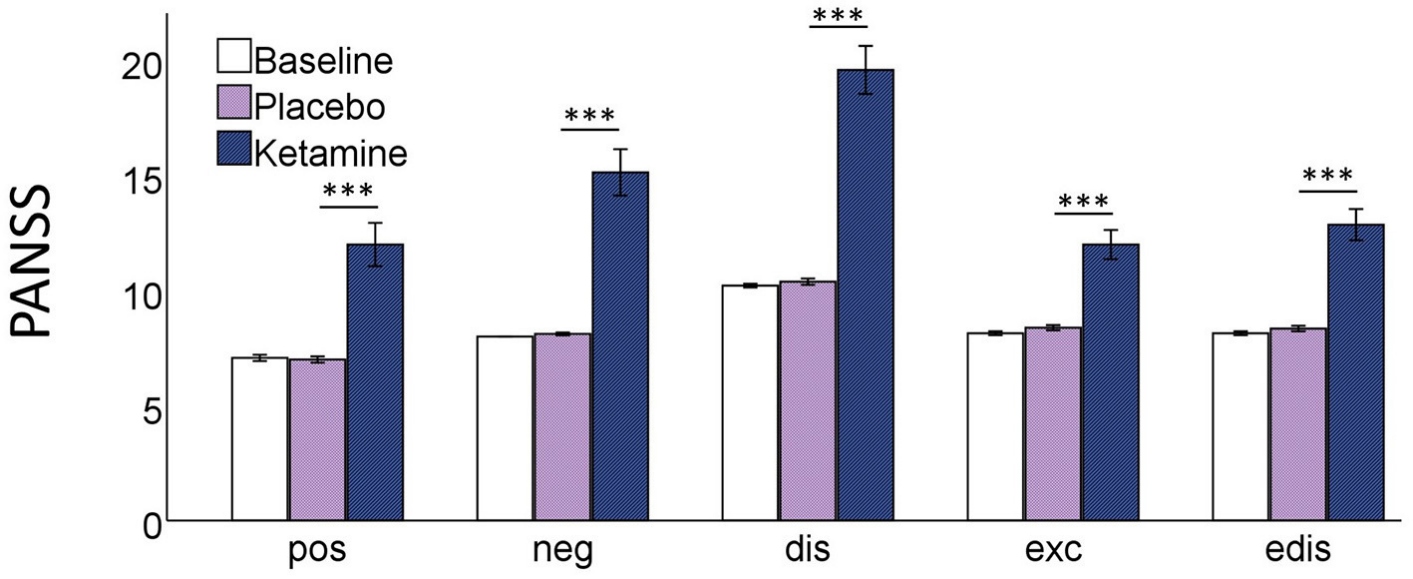
Bar charts of the mean values of the five PANSS subscales; **pos** positive symptoms, **neg** negative symptoms, **dis** disorganisation, **exc** excitement, **edis** emotional distress; with error bars representing ±1 standard errors of the mean (***p < 0.001).

**Figure 2:**
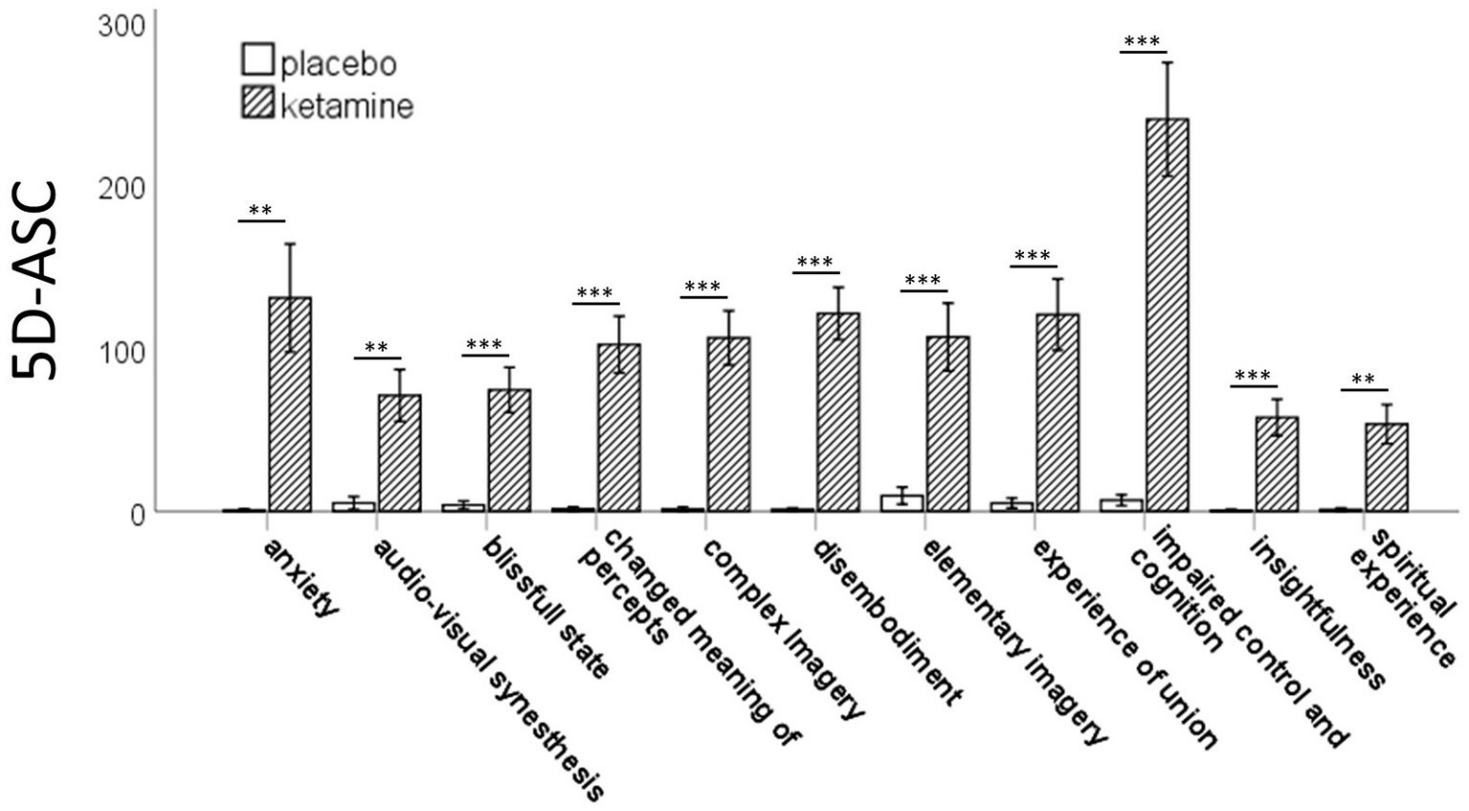
Bar charts of the mean values of the 11 subscales of the 5D-ASC-scale with error bars representing ±1 standard errors of the mean (**p < 0.01;***p < 0.001).

The RM-ANOVA showed significant effects of session (baseline, placebo, ketamine) on all subscales of the PANSS (positive symptoms: *F*_(2,76)_ = 30.49, *p* < 0.001; negative symptoms: *F*_(2,76)_ = 54.18, *p* < 0.001; disorganization symptoms: *F*_(2,76)_ = 90.18, *p* < 0.001; excitement: *F*_(2,76)_ = 38.80, *p* < 0.001; emotional distress: *F*_(2,76)_ = 51.74, *p* < 0.001). All five factors were significantly increased after ketamine administration compared to placebo administration and baseline values according to *post-hoc t*-tests. There were no significant differences between the baseline and the placebo session (Figure 1). The paired-samples *t*-test showed significant increases (*p* < 0.01) in all 11 subscales of the 5D-ASC-questionnaire (Figure 2).

### Ketamine-Induced Changes of Resting-State Oscillations

**Figure 3:**
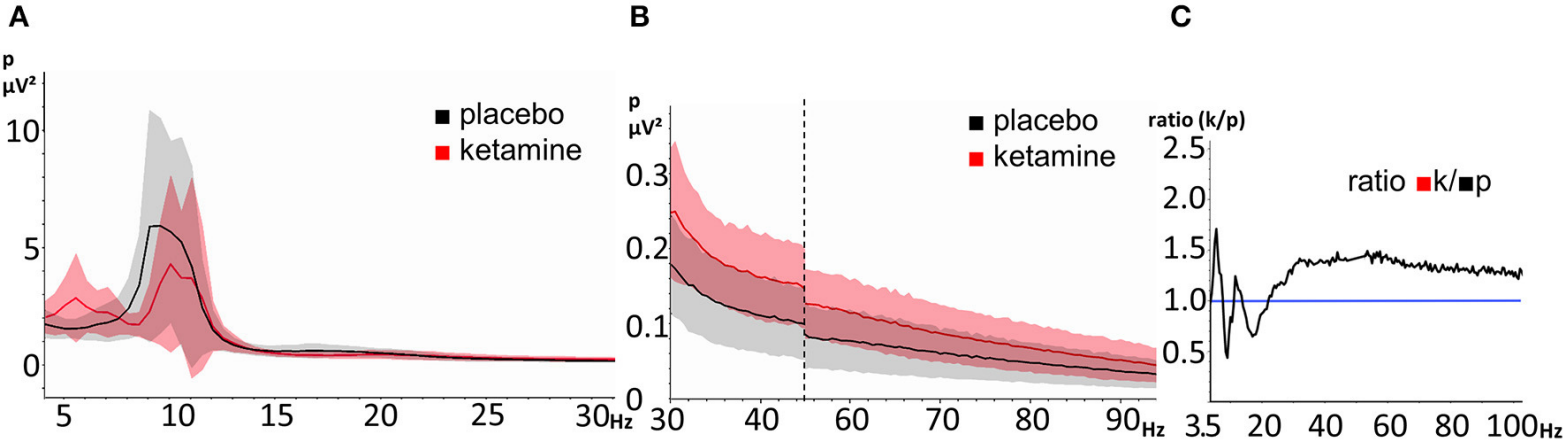
EEG resting-state power spectra during the ketamine and placebo condition. The shaded areas represent the standard deviation (red = ketamine, gray = placebo). **(A)** frequency-range 3,5 Hz- 30Hz, **(B)** frequency-range 30–100 Hz (the vertical dashed line indicates scale-break between 45 and 55 Hz) and **(C)** ratio of power between ketamine (numerator) and placebo (denominator) (range: 3.5–100 Hz). The horizontal line represents a ratio of 1.

The mean power was plotted as a function of frequency for both the placebo and the ketamine condition (Figures 3A,B). The resting-state oscillatory power was elevated under the influence of ketamine for the frequency ranges of 4.5–8 Hz, 12–14 Hz, and above 22,5 Hz (>1 in Figure 3C).

### Scalp-Level Resting-State EEG Analysis

**Figure 4:**
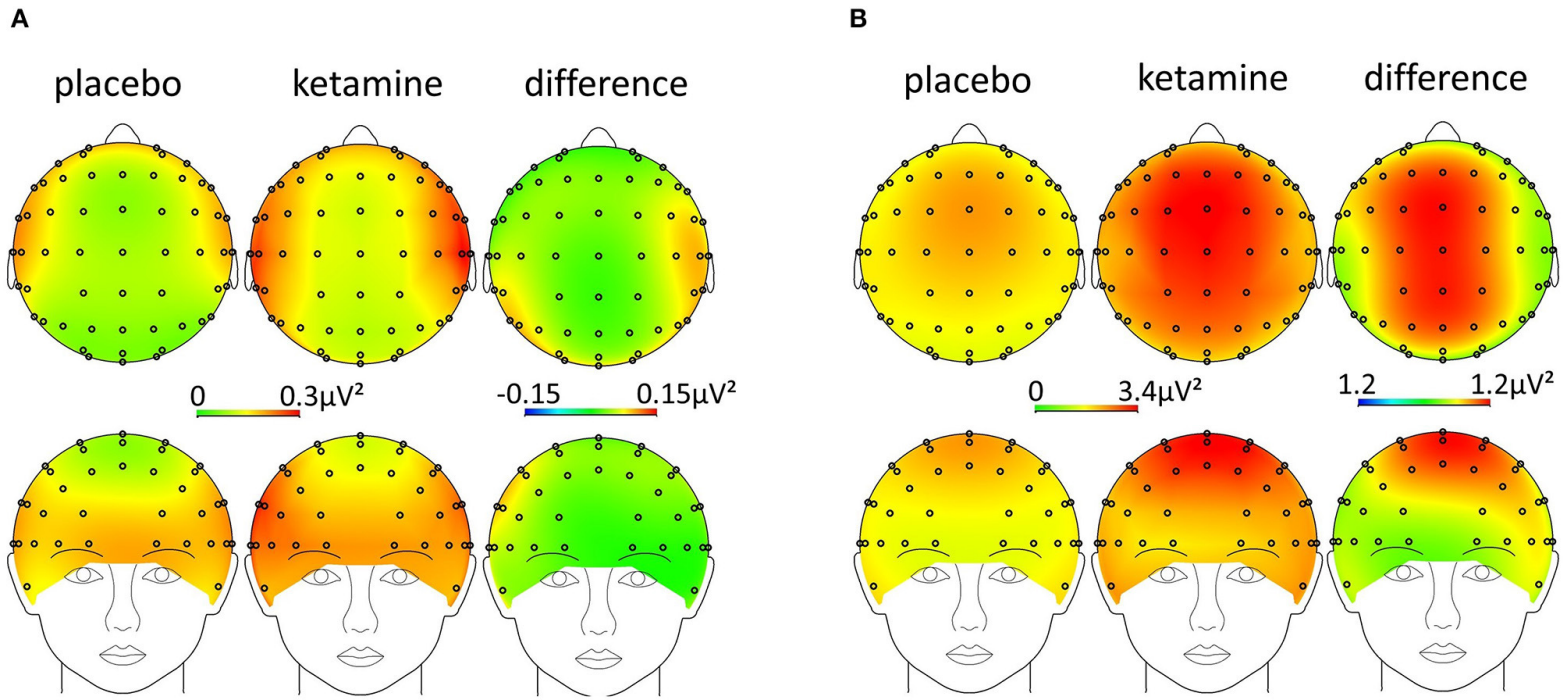
Scalp-topography [ketamine, placebo, and difference (ketamine-placebo)] in the **(A)** gamma- (30–40 Hz) and **(B)** theta-band (4–8 Hz).

An RM-ANOVA was performed in order to examine the interaction of the factors condition (ketamine, placebo), region on the scalp (frontal, temporo-central, occipital), and laterality (left, middle, right) regarding resting-state gamma- (30–40Hz) and theta-power (4–8Hz). There was a significant three-way interaction of condition x region x laterality on gamma-power, *F*_(2.6,53.7)_ = 6.3, *p* = 0.002, ε = 0.64. The Bonferroni-corrected *post-hoc* paired sample *t*-tests showed a significant increase in resting-state gamma-power under the influence of ketamine in all regions, except for the frontal left and right region (see Table 1 and Figure 4A). The same approach for the theta-band (4–8 Hz) revealed a significant two-way interaction of condition x laterality on theta power *F*_(1.2,24.7)_ = 43.9, *p* < 0.001, ε = 0.50. The Bonferroni-corrected *post-hoc* paired sample *t*-tests showed a significant increase in resting-state theta-power under the influence of ketamine in the middle regions (see Table 1 and Figure 4B).

**Table 1:** Mean power (in μV^2^) (± SD) in the gamma γ (30–40Hz) and theta θ (4–8 Hz) frequency range for different regions, laterality and condition (***p < 0.001, **p < 0.01, *p < 0.05)].

	**Left**		**Middle**		**Right**	
	**Placebo**	**Ketamine**	**Placebo**	**Ketamine**	**Placebo**	**Ketamine**
Frontal θ	1.57 ± 0.39	2.22 ± 0.70^**^	2.10 ± 0.72	3.32 ± 1.31^***^	1.59 ± 0.42	2.05 ± 0.63^**^
Temporo-central θ	1.70 ± 0.52	2.20 ± 0.95	1.95 ± 0.56	2.87 ± 1.11^***^	1.72 ± 0.59	2.18 ± 0.90
Posterior θ	1.53 ± 0.57	1.94 ± 0.94	1.69 ± 0.58	2.77 ± 1.19^***^	1.51 ± 0.60	1.89 ± 0.96
Frontal γ	0.174 ± 0.072	0.200 ± 0.065	0.093± 0.041	0.139 ± 0.053^**^	0.167 ± 0.062	0.213 ± 0.067
Temporo-central γ	0.182 ± 0.084	0.248 ±0.094^*^	0.118± 0.051	0.163 ± 0.054^*^	0.171 ± 0.073	0.252 ± 0.107^*^
Posterior γ	0.098 ± 0.063	0.200 ± 0.086^***^	0.086± 0.050	0.119 ± 0.036^*^	0.083 ± 0.044	0.176 ± 0.092^***^

For the reference-free current source density (CSD) in the gamma-band (30–40 Hz), the RM-ANOVA revealed a significant two-way interaction of condition x region *F*_(2,47)_ = 4.1, *p* = 0.024. For the CSD in the theta-band (4–8 Hz), the RM-ANOVA revealed two significant two-way interactions of condition x region *F*_(1.5,32.0)_ = 4.026, *p* = 0.0373, ε = 0.76 and condition x laterality *F*_(2,42)_ = 3.5, *p* = 0.0381. The Bonferroni-corrected *post-hoc* paired sample *t*-tests showed a significant increase in resting-state gamma-CSD, though only in the posterior right region (see Table 2).

**Table 2:** Mean CSD (in μV/m^2^) (± SD) in the gamma γ (30–40Hz) and theta θ (4–8 Hz) frequency range for different regions, laterality and condition (**p < 0.01).

	**Left**		**Middle**		**Right**	
	**Placebo**	**Ketamine**	**Placebo**	**Ketamine**	**Placebo**	**Ketamine**
Frontal θ	0.33 ± 0.12	0.33 ± 0.16	0.37 ± 0.18	0.40 ± 0.23	0.34 ± 0.15	0.30 ± 0.13
Temporo-central θ	0.27 ± 0.11	0.28 ± 0.12	0.25 ± 0.10	0.27 ± 0.11	0.26 ± 0.08	0.26 ± 0.13
Posterior θ	0.39 ± 0.25	0.32 ± 0.13	0.30 ± 0.10	0.30 ± 0.15	0.41 ± 0.23	0.30 ± 0.15
Frontal γ	0.127 ± 0.047	0.121 ± 0.056	0.077 ± 0.034	0.075 ± 0.023	0.136 ± 0.061	0.129 ± 0.065
Temporo-central γ	0.121 ± 0.071	0.112 ± 0.061	0.061 ± 0.025	0.064 ± 0.042	0.094 ± 0.056	0.116 ± 0.064
Posterior γ	0.104 ± 0.046	0.120 ± 0.055	0.059 ± 0.016	0.063 ± 0.029	0.085 ± 0.035	0.117 ± 0.055^**^

### Source-Level Gamma-Band and Theta-Band Connectivity Changes

**Figure 5:**
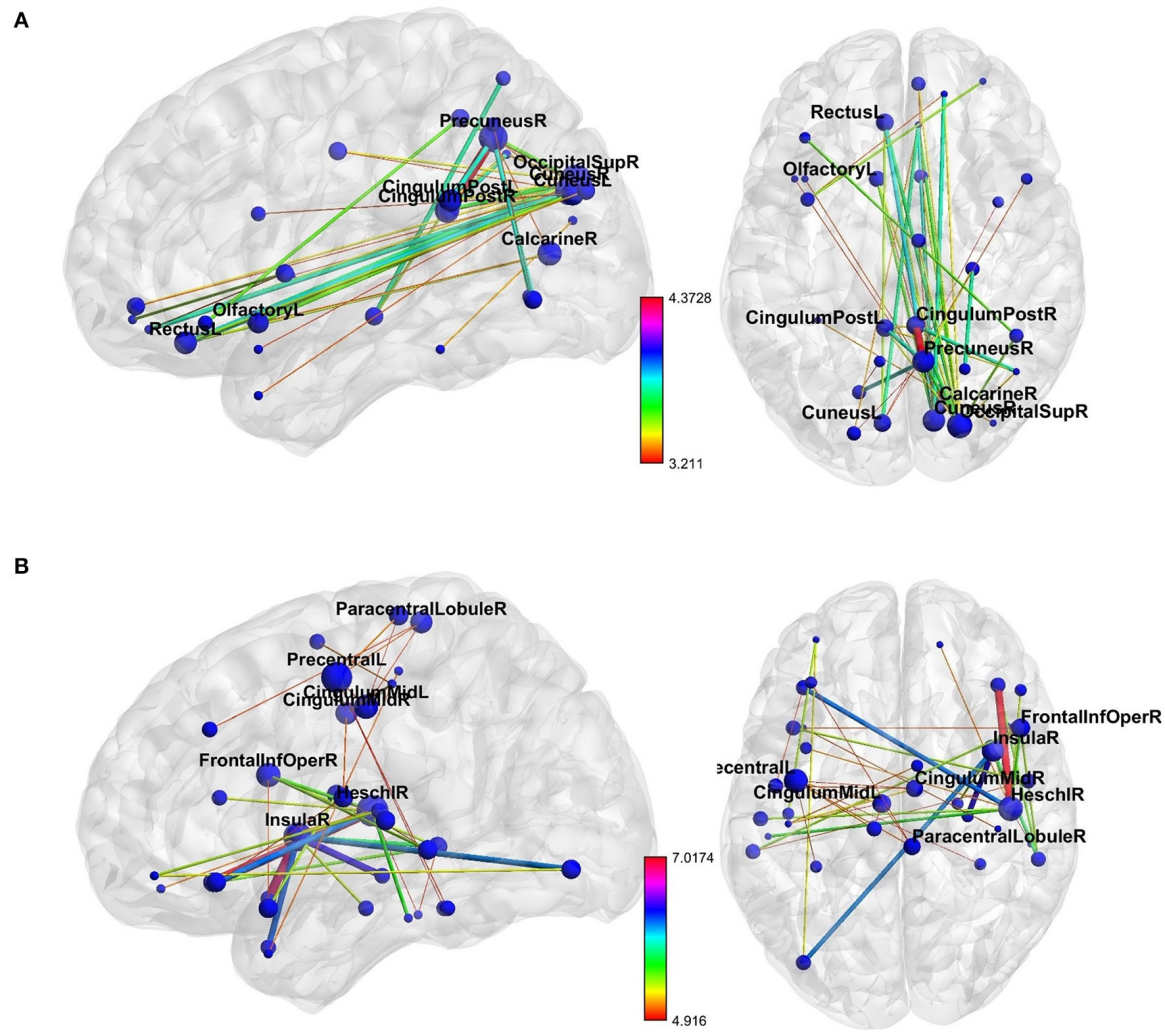
The networks of increased resting-state **(A)** gamma- and **(B)** theta-band connectivity during the ketamine compared to placebo administration. The size of each node represents its number of connections within the network (degree). The thickness and color of connections represent the *t*-value. Labels are provided for nodes with at least three connections. As shown in Table 3, these nodes are involved in more than 50% of total interactions within the theta-network and more than 70% of total interactions within the gamma-network. The figure was created using BrainNet Viewer (http://www.nitrc.org/projects/bnv/).

**Figure 6:**
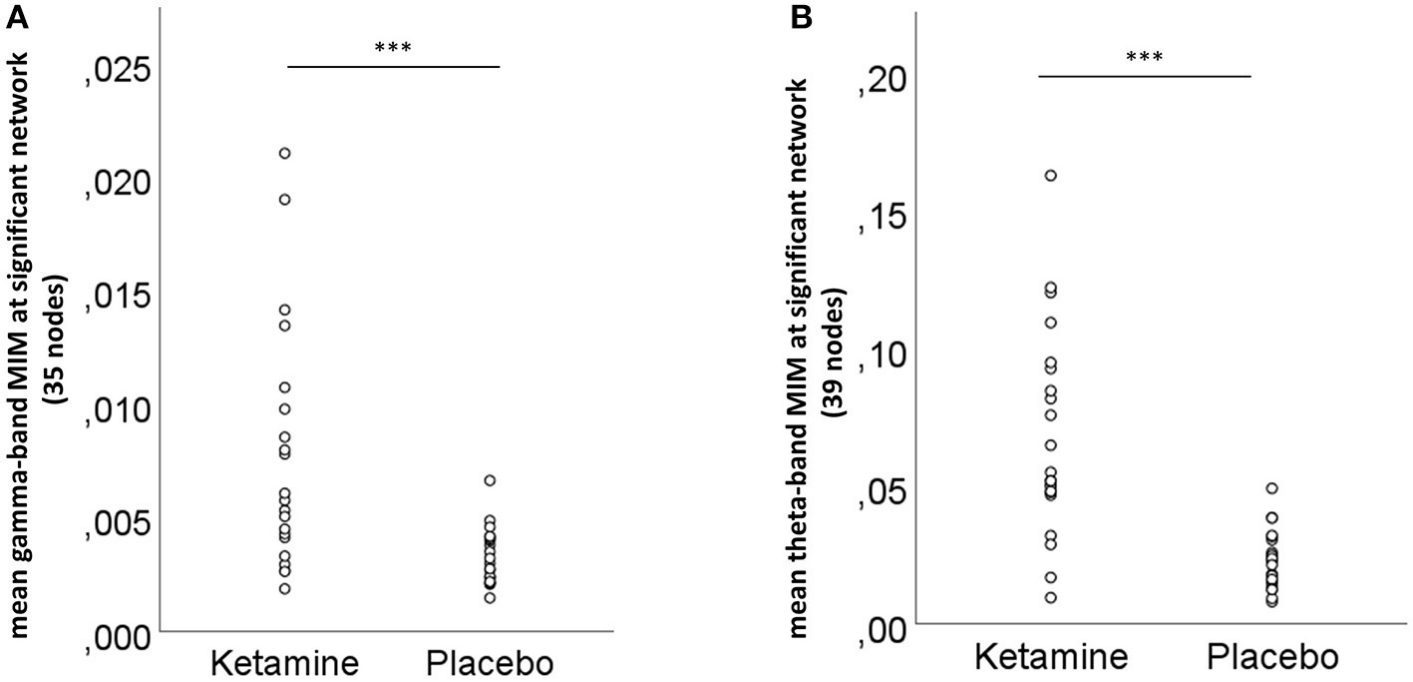
Mean **(A)** gamma- and **(B)** theta-band connectivity across the nodes of the significant networks for the ketamine and placebo condition, ***p < 0.001 (Bonferroni-corrected).

NBS revealed a network displaying increased connectivity in the gamma-band during ketamine administration compared to placebo with the imaginary coherence-based *Multivariate Interaction Measure* (MIM) being the measure of connectivity (threshold *t* = 3.2, corr. *p* = 0.0034). This network comprised 45 connections involving midline regions, the cuneus, the precuneus and the bilateral posterior cingulate cortex (Table 3, Figure 5A). NBS also revealed a network displaying increased MIM-connectivity in the theta-band during ketamine administration compared to placebo (threshold *t* = 4.8, corr. *p* < 0.0001). This network comprised 39 connections involving the Heschl gyrus and midline regions, the insula, and the middle cingulate cortex (Table 3, Figure 5B). Mean connectivity values within the networks mentioned above are displayed in Figure 6. There was a significant increase in the MIM-activity in both the respective gamma-network under the influence of ketamine (M = 0.0077, SD = 0.0054) compared to placebo (M = 0.00327, SD = 0.0012); *t*_(21)_ = −6.194, *p* < 0.001; and in the theta-network under the influence of ketamine (M = 2.4, SD = 0.9) compared to placebo (M = 1.7, SD = 0.5), *t*_(21)_ = −3.869, *p* < 0.001.

**Table 3:** Brain regions included in the networks of increased gamma and theta connectivity under the influence of ketamine compared to placebo condition, in the order of their degree (number of connections) within the network.

**40 Hz**			**6 Hz**		
**Region**	**Hemisphere**	**Degree**	**Region**	**Hemisphere**	**Degree**
Superior occipital gyrus	Right	14	Heschl gyrus	Right	10
Cuneus	Right	9	Precentral gyrus	Left	9
Precuneus	Right	9	Insula	Right	7
Posterior cingulate cortex	Right	5	Middle cingulate cortex	Left	4
Posterior cingulate cortex	Left	4	Inferior frontal operculum	Right	4
Cuneus	Left	4	Superior temporal gyrus	Right	4
Gyrus rectus	Left	4	Middle cingulate cortex	Right	3
Gyrus rectus	Right	4	Paracentral lobule	Right	3
Angular gyrus	Right	3	Inf. frontal gyrus, orbital part	Left	2
Calcarine sulcus	Right	3	Inf. frontal gyrus, orbital part	Right	2
Superior frontal gyrus, orbital part	Right	3	Middle orbitofrontal cortex	Left	2
Olfactory gyrus	Left	3	Inferior occipital gyrus	Left	2
Middle cingulate cortex	Right	2	Paracentral lobule	Left	2
Medial orbitofrontal cortex	Right	2	Rolandic operculum	Left	2
Hippocampus	Right	2	Rolandic operculum	Right	2
Insula	Left	2	Temporal pole, superior temp. gyrus	Left	2
Lingual gyrus	Left	2	Temporal pole, superior temp. gyrus	Right	2
Superior occipital gyrus	Left	2	Middle temporal gyrus	Left	2
Olfactory gyrus	Right	2	Superior temporal gyrus	Left	2
Inferior parietal lobule	Right	2	Inf. frontal gyrus, pars triangularis	Right	1
Inferior frontal operculum	Right	1	Middle frontal gyrus	Left	1
Inferior frontal operculum	Left	1	Superior frontal gyrus, orbital part	Right	1
Middle orbitofrontal cortex	Right	1	Fusiform gyrus	Left	1
Insula	Right	1	Fusiform gyrus	Right	1
Lingual gyrus	Right	1	Heschl gyrus	Left	1
Superior parietal lobule	Right	1	Hippocampus	Right	1
Precuneus	Left	1	Insula	Left	1
Temporal pole, middle temp. gyrus	Left	1	Parahippocampal gyrus	Right	1
Temporal pole, sup. temp. gyrus	Left	1	Supplementary motor area	Right	1
			Inferior temporal gyrus	Right	1
			Middle temporal gyrus	Right	1

### Association Between Neurophysiological Measures and Psychopathological Variables

**Figure 7:**
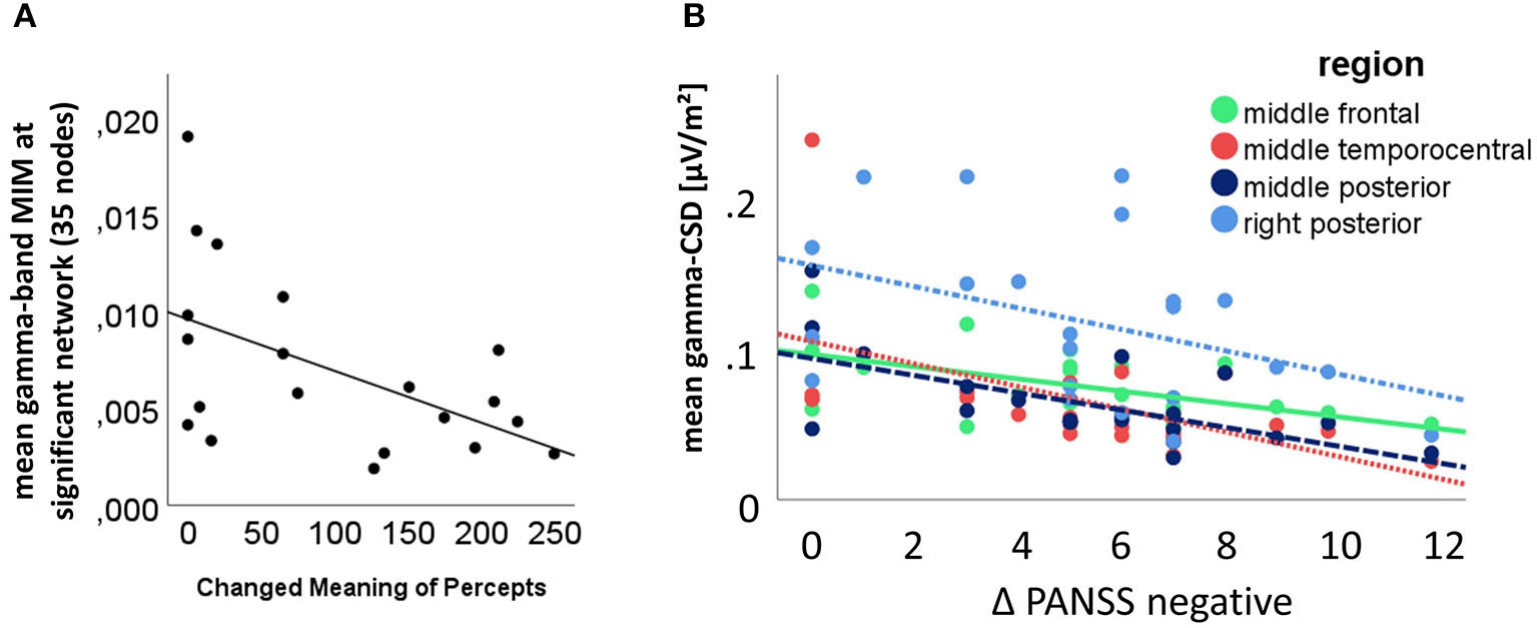
**(A)** Significant multivariate analysis of variance result, with the mean connectivity within the significant 45 connections involving gamma-network as dependent variable and the 5D-ASC *changed meaning of percepts* scale as predictor (bivariate Pearson's *r* = −0.534; *n* = 21; *p* = 0.008); **(B)** Correlation between the CSD in the significant regions as revealed by the multivariate analysis of variance results and *post-hoc t*-testing; the bivariate correlations between PANSS-negative factor increase and the middle frontal region (solid green line) (Pearson's *r* = −0.541; *n* = 22; *p* = 0.005), the middle temporo-central region (densely dotted red line) (Pearson's *r* = −0.562; *n* = 22; *p* = 0.003), the posterior middle region (dashed dark blue line) (Pearson's *r* = −0.627; *n* = 22; *p* = 0.001) and the right posterior region (dashdotted light blue line) (Pearson's *r* = −0.412; *n* = 22; *p* = 0.028) were Bonferroni corrected.

The multiple linear regression analysis indicated that only the 5D-ASC *changed meaning of percepts* factor of the 5D-ASC under the influence of ketamine significantly predicted the mean activity within the significant gamma-band network [*F*_(1,19)_ = 5.304; *p* = 0.037], while other factors were not significantly associated when controlling for the effect of all other symptoms. There was also a significant correlation between the *changed meaning of percepts* score and the mean network strength in the *post-hoc* bivariate correlation analysis (Pearson's *r* = −0.534; *n* = 21; *p* = 0.008) (Figure 7). Another multiple linear regression analysis revealed that the resting-state gamma-CSD under ketamine was a significant predictor of the PANSS negative factor when entering all 9 regions and all PANSS-subscales in the model. The model showed significant results for associations between the resting-state gamma-CSD in the middle temporal-central area, the frontal left and middle area, the left and middle temporo-central area, and the middle posterior area and PANSS negative symptoms score *F*_(1,20)_ = 5.3–13.283, *p* ≤ 0.036. There were no significant associations between the other PANSS subscales and CSD-scores. A negative bivariate correlation between the PANSS negative symptoms score and the mean CSD was confirmed in 4 of the 6 regions from the previous analysis after correlation Bonferroni correction (see Figure 7). RS-TBO measures were not associated with schizophrenia-like symptoms.

## Discussion

In this placebo-controlled drug-challenge 64-channel-resting-state-EEG study with a crossover design, we showed that ketamine increases local resting-state synchrony (power and current source density) in the gamma- and theta-frequency range, as well as the long-range oscillatory coupling (connectivity) within frequency-specific networks. The gamma network consisted of occipital, midline, and frontal regions, while the theta network involved the Heschl gyrus, frontal and temporal regions. The current source density (CSD) in the gamma-band was negatively correlated with the PANSS negative factor increase under ketamine. The strength of the significant gamma-networks was associated with the decreased *changed meaning of percepts* self-rating scale of the 5D-ASC. Ketamine caused psychopathological changes similar to those seen in patients with schizophrenia, which was assessed by PANSS and the 5D-ASC. These results are in line with our hypothesis of a ketamine-induced schizophrenia-like alteration of behavior, perception, and resting-state oscillations due to dysfunction of the NMDAR. The findings argue for the proposed dysfunction of the glutamatergic system as a core pathophysiological mechanism of schizophrenia.

The observation of increased resting-state gamma-band oscillations (RS-GBO) under the influence of ketamine parallels observations made in patients with schizophrenia (11–13, 61). As elaborated in the introduction, the generation of GBO involves a neural microcircuit consisting of pyramidal cells, fast-spiking inhibitory GABA-releasing PV^+^-interneurons (23), and SST^+^-interneurons (28). The NMDA-receptors not only on the interneurons but also on the pyramidal cells seem to play an essential role in maintaining this circuitry involved in GBO-formation (62, 63). Our results support the importance of the glutamatergic neurotransmission as the underlying mechanism of disturbed GBO in schizophrenia. They are in line with observations of increased resting-state connectivity in the gamma-band in patients with schizophrenia (20, 64). Di Lorenzo et al. (64) observed significantly higher connectivity between right occipital-prefrontal, occipital-parieto-temporal, and occipital-cingulate ROI-pairs, while Andreou et al. found increased connectivity in bilateral frontal and insula regions and left temporal-midline regions. This study's connectivity patterns with an involvement of the right occipital and cingulate area parallel the findings from schizophrenia patients and are consistent with other RS-ketamine-studies, which found increased RS-GBO (42–44, 65). The gamma network involved areas relevant for auditory–spatial processing, working memory, attention and regions of the default mode network, which have been described to be affected in schizophrenia (66–69). Accordingly, the present results may link aberrant RS-gamma-band connectivity, glutamatergic deficits, and core clinical symptoms of schizophrenia.

The *changed meaning of percepts* factor of the 5D-ASC, which was negatively correlated with the RS-GBO-network, consisted of three items: 1. “Everyday things gained a special meaning.” 2. “Things around me had a new, strange meaning for me.” 3. “Objects around me engaged me emotionally much more than usual.” These items resemble the delusional mood, or “Wahnstimmung,” first described by Klaus Conrad in 1958 and Mishara (70), which is typically seen in the early stages of schizophrenia, before actual delusions or other positive symptoms evolve. Interestingly, Andreou et al. also showed a negative correlation between a resting-state gamma-network in patients with schizophrenia and positive/disorganization measured by the PANSS (20). Consistently, a resting-state ketamine-MEG-study showed a negative correlation between the source-power in the right hippocampus, which was also part of the network in this study, and positive symptoms (41). While these results resemble patients with a first-episode of schizophrenia, a comparison among different illness stages would be interesting. In a recent study, Grent-'t-Jong et al. (13) explored RS-GBOs in participants meeting clinical high-risk criteria, first-episode patients, and patients with chronic schizophrenia (13). Interestingly, in the chronic schizophrenia-group, PANSS-scores were correlated with a reduction of gamma-band power, especially in the gamma-band range between 30 and 46 Hz. This is also in line with our findings of a negative correlation between RS-CSD-GBO and negative symptoms. Grent-'t-Jong et al. (13) findings of a reduction of RS-power between a first-episode and chronic-schizophrenia emphases a higher consistency between the ketamine-model of schizophrenia and earlier stages of the disease.

Consistent with our results, increased resting-state theta-band oscillations (RS-TBO) in schizophrenia patients have been observed in most MEG and EEG studies (17). As some animal studies suggest, the underlying mechanism of the generation of TBO is believed to depend on glutamatergic neurotransmission via the NMDAR (71), which is supported by this study. Several studies also showed increased theta-band connectivity patterns in patients with schizophrenia (15, 22, 64). Andreou et al. observed increased connectivity in frontal, temporal, parietal, and midline areas using the same *Multivariate Interaction Measure* (MIM) utilized in this study, making the two studies highly comparable. Our study showed a substantial number of connections leading to the precentral left gyrus comparable to the study of Andreou et al. In contrast to medicated patients suffering from their first episode of schizophrenia, healthy subjects receiving ketamine had additional right hemisphere regions involved in the network. A possible explanation would be an influence of dopaminergic drugs on the networks in the schizophrenia studies or an emerging dysconnectivity throughout the disease. The number of resting-state studies exploring drug-naïve resting-state oscillatory patterns before and after therapy is still limited (72). Future studies with drug-naïve patients and studies within the ketamine model of schizophrenia could involve modulation by dopaminergic substances. The increase of theta-band connectivity in this study involved a distinct network comprising areas relevant for auditory processing (73), motor-function (74), representations of the self (75), areas relevant to language processing (76) and thought disorders (77), all of them highly relevant for schizophrenia.

As mentioned before, this study was part of a larger project with the same subjects. During the same session, subjects were examined not only with respect to resting-state oscillations, but also regarding other neurophysiological biomarkers of schizophrenia within the theta band (mismatch negativity [MMN]) (45), and gamma-band; both evoked GBO (47) and interhemispheric gamma-connectivity between auditory cortices (46). Interestingly the measures of GBO within the ketamine-model of schizophrenia showed diverse oscillatory patterns, strongly resembling schizophrenia patients: (1) the RS-GBO and TBO were elevated as shown in this study, (2) sensory-evoked gamma-band responses were reduced (47), (3) interhemispheric-coupling was increased (46) and (3) MMN, that reflects activity primarily within the theta (4–7Hz) frequency band (78), was reduced (46). These contrasting results do not only parallel characteristic findings seen in patients with schizophrenia (79–82) but are also reinforced by basic research studies. In an optogenetic mouse model, a light-activation of glutamate PV+-interneurons, resulted in both elevated resting-state GBO and reduced evoked gamma-oscillations (26). Taking all the above into account, the ketamine-model of schizophrenia can help to identify and develop potential biomarkers (treatment response prediction biomarkers and therapeutic monitoring biomarkers) that are connected to molecular targets for glutamatergic treatment options and to clinical symptoms (83).

This study both explored the scalp-CSD and the power, as both measures are reported frequently in schizophrenia studies. However, the CSD has several advantages and is suggested as a superior measure of RS-scalp analysis for several reasons (84). As a reference-free method, it avoids difficulties related to reference-dependent EEG measures, it has a sharper topography by reducing the negative impact of volume conduction and more closely represents underlying neuronal activity (85). In this study, scalp level gamma- and theta RS-power-changes have been observed in several regions, while CSD only revealed a significant activity change in the posterior right region. This might be due to the reduction of volume conduction. As only PANSS-negative symptoms were correlated with CSD-measures of scalp RS-gamma-activity, not with power changes, it can be reasoned that CSD-measures represent brain activity related to clinical symptoms of schizophrenia. We therefore strongly support the reporting of scalp-CSD.

A limitation of this study, like others, was that the ketamine-effects could have caused an unblinding. While controlling with NaCl has certain advantages on the EEG level, controlling with another drug with psychotomimetic actions could help overcome this bias in future studies. Using a dopaminergic drug as control could also help compare the glutamate and dopamine effects on the same neurophysiological measures. The issue of possible false-positive findings arising from the multiplicity of analyses used in this study had to be addressed. Accordingly, we used Bonferroni-corrected *post-hoc t*-tests after preselecting the data with RM-ANOVA or linear regression analysis and the non-parametric statistical method implemented in NBS to deal with the multiple comparisons problem on a graph.

The sample studied only consisted of highly educated male volunteers with a tight age range of 20–32 years. While the age of the participants is reasonable in view of the typical age of onset for schizophrenia (86), further studies should include female subjects.

On the scalp level, the statistical method used only showed robust effects. Other methods like the non-parametric statistical testing and cluster-based analysis approach could have revealed more subtle changes (87).

In conclusion, we report ketamine-induced increases of local resting-state synchrony within the gamma and theta band and increased long-range oscillatory coupling, measured by the imaginary coherence-based *Multivariate Interaction Measure* (MIM), in healthy subjects. In the source-space, network-based statistics identified two significant cortical networks within 80 predefined cortical regions. The gamma-network consisted of midline regions, the cuneus, the precuneus, and the bilateral posterior cingulate cortices, while the theta-band network involved the Heschl gyrus, midline regions, the insula, and the middle cingulate cortex. This study showed the relevance of local and network synchrony in the gamma-band for schizophrenia-like negative symptoms and delusional mood-like symptoms. The schizophrenia-like gamma- and theta-oscillatory fingerprints under the influence of ketamine, the crucial role of glutamatergic neurotransmission in the generation of gamma- and theta- oscillations, and its association with clinical symptoms, all strongly support the glutamate hypothesis of schizophrenia. This study establishes a link between preclinical studies and resting-state studies of patients suffering from schizophrenia. The results argue for an important role of glutamate for the balance in excitatory and inhibitory activity (E/I-balance) within local and long-range oscillatory patterns in the gamma and theta frequency range in schizophrenia. Those patterns could serve as a biomarker for the response of patients to glutamatergic drugs or help to test the eligibility for glutamatergic interventions. The ketamine model of schizophrenia provides a tool for testing the influence of glutamatergic or dopaminergic substances or stimulation settings like rTMS on brain networks and their influence on the clinical symptoms of schizophrenia.

## Data Availability Statement

The datasets presented in this article are not readily available due to information that could compromise the privacy of research participants. Requests to access the datasets should be directed to s.curic@uke.de.

## Ethics Statement

The study involved human participants and was reviewed and approved by Ethics Committee of the Medical Association Hamburg. The participants provided their written informed consent to participate in this study.

## Author Contributions

SC, CM, GL, and CA designed the study and wrote the protocol. MH, ST, NP, SS, and JR managed literature searches and data acquisition. SC, CA, and GN conducted electroencephalographic and statistical analyses. SC and CA interpreted findings. SC wrote the first draft of the manuscript. All authors contributed to and have approved the final manuscript.

## Conflict of Interest

The authors declare that the research was conducted in the absence of any commercial or financial relationships that could be construed as a potential conflict of interest.
