# Supplementary material for: Ketamine Alters Functional Gamma and Theta Resting-State Connectivity in Healthy Humans: Implications for Schizophrenia Treatment Targeting the Glutamate System
Source: Front Psychiatry. 2021 Jun 10;12:671007. doi: 10.3389/fpsyt.2021.671007 (PMC8222814; doi:10.3389/fpsyt.2021.671007)
Supplement: Supplementary file 1 [file Table_1.DOCX]

Supplementary Material

| Table S1 Coordinates of the 80 cortical points used for connectivity analyses. | | | | | | | |
| --- | --- | --- | --- | --- | --- | --- | --- |
|  | | Left hemisphere | | | Right hemisphere | | |
|  |  | x | y | z | x | y | z |
| Medial orbitofrontal cortex | | -5 | 55 | -5 | 5 | 50 | -5 |
| Middle orbitofrontal cortex | | -30 | 50 | -10 | 30 | 55 | -10 |
| Superior frontal gyrus, medial part | | -5 | 50 | 30 | 10 | 50 | 30 |
| Superior frontal gyrus, orbital part | | -20 | 50 | -15 | 15 | 50 | -15 |
| Anterior cingulate cortex | | -5 | 35 | 15 | 5 | 35 | 15 |
| Middle frontal gyrus | | -35 | 35 | 35 | 35 | 35 | 35 |
| Superior frontal gyrus | | -20 | 35 | 40 | 20 | 30 | 45 |
| Gyrus rectus | | -5 | 35 | -20 | 5 | 35 | -20 |
| Inferior frontal gyrus, orbital part | | -35 | 30 | -10 | 40 | 30 | -10 |
| Inferior frontal gyrus, pars triangularis | | -45 | 30 | 15 | 45 | 30 | 15 |
| Inferior frontal operculum | | -50 | 15 | 20 | 50 | 15 | 20 |
| Olfactory gyrus | | -5 | 15 | -10 | 5 | 15 | -10 |
| Temporal pole, middle temporal gyrus | | -35 | 15 | -35 | 45 | 15 | -30 |
| Temporal pole, superior temporal gyrus | | -40 | 15 | -20 | 45 | 15 | -15 |
| Insula | | -40 | 10 | 0 | 40 | 10 | 0 |
| Supplementary motor area | | -5 | 5 | 60 | 10 | 0 | 60 |
| Precentral gyrus | | -40 | -5 | 50 | 40 | -10 | 50 |
| Rolandic operculum | | -50 | -10 | 15 | 50 | -5 | 15 |
| Middle cingulate cortex | | -5 | -15 | 40 | 5 | -10 | 40 |
| Parahippocampal gyrus | | -20 | -15 | -20 | 20 | -15 | -20 |
| Heschl gyrus | | -45 | -20 | 10 | 45 | -15 | 10 |
| Hippocampus | | -25 | -20 | -10 | 25 | -20 | -10 |
| Superior temporal gyrus | | -55 | -20 | 5 | 55 | -20 | 5 |
| Paracentral lobule | | -5 | -25 | 70 | 5 | -30 | 70 |
| Postcentral gyrus | | -45 | -25 | 50 | 40 | -25 | 55 |
| Inferior temporal gyrus | | -50 | -30 | -25 | 55 | -30 | -20 |
| Supramarginal gyrus | | -55 | -35 | 30 | 55 | -30 | 35 |
| Middle temporal gyrus | | -55 | -35 | 0 | 55 | -35 | 0 |
| Fusiform gyrus | | -30 | -40 | -20 | 35 | -40 | -20 |
| Posterior cingulate cortex | | -5 | -45 | 25 | 5 | -45 | 20 |
| Inferior parietal lobule | | -45 | -45 | 45 | 45 | -45 | 50 |
| Precuneus | | -10 | -55 | 50 | 10 | -55 | 45 |
| Superior parietal lobule | | -25 | -60 | 60 | 25 | -60 | 60 |
| Angular gyrus | | -45 | -65 | 40 | 40 | -60 | 40 |
| Lingual gyrus | | -15 | -70 | -5 | 15 | -65 | -5 |
| Calcarine sulcus | | -10 | -80 | 10 | 15 | -75 | 10 |
| Cuneus | | -5 | -80 | 25 | 15 | -80 | 30 |
| Inferior occipital gyrus | | -35 | -80 | -10 | 35 | -80 | -10 |
| Middle occipital gyrus | | -30 | -80 | 15 | 35 | -85 | 20 |
| Superior occipital gyrus | | -20 | -85 | 30 | 20 | -80 | 30 |
| Table S2 electrode locations selected for electrode pools |  |  |  |  |  |  |  |
|  |  |  |  |  |  |  |  |

|  | laterality | | |
| --- | --- | --- | --- |
| region | left | middle | right |
| frontal | AF7  F3  F5  F7 Fp1 | F2 FC1 FC2 FCz  Fz | AF8 F4  F6  F8 Fp2 |
| temporo-central | C5 CP5 FC5 FT7  T7 TP7 | C1  C2  C3  C4  Cz | C6  CP6 FC6 FT8 T8 TP8 |
| posterior | O1  P3  P5  P7 PO3 PO9 | CP1 CP2 CPz P1  P2  Pz | O2  P4  P6  P8 PO10 PO4 |

| Table S3 Sociodemographic Characteristics of Participants at Baseline | |
| --- | --- |
| Age  Gender  Male | 25 ± 2.6 (range:20-32)  100% |
| Education in years | 17.8 ± 1.9 (range: 14-21) |
| Verbal IQ | 113 ± 6,24 (range: 99-125) |
| Education of father in years | 16.7 ± 1.9 (range: 13-19) |
| Education of mother in years | 18.1± 2.0 (range: 13-22) |
| Nicotine consumption  Smoker  Non-smoker | 34,8 %  65,2 % |

Network-based statistic

The network-based statistic (NBS) (Zalesky, Fornito, & Bullmore, 2010) was implemented in the present study to identify networks resembling schizophrenia under ketamine in healthy subjects: For each of the 3160 pairwise connections, a (one-sided) t-test statistic was computed between the imaginary coherence-based multivariate interaction measure (MIM) values of the ketamine and placebo group separately. The threshold (t=3.2 for the gamma and 4.8 for the theta condition, corresponding to an uncorrected p-value of 0.005) was applied to construct a set of suprathreshold links. Clusters identified the connected graph components. Permutation testing (5000 permutations) was subsequently used to determine the significance value of each connected component based on its topological extent, corrected for multiple comparisons. These analyses were conducted using the open-source toolbox NBS Connectome v1.2 (http://www.nitrc.org/projects/nbs). Because contrasts were conducted in two directions, the significance level was set to 0.025.

REFERENCES

Nichols, T. E., & Holmes, A. P. (2002). Nonparametric permutation tests for functional neuroimaging: a primer with examples. *Human brain mapping, 15*(1), 1-25.

Zalesky, A., Fornito, A., & Bullmore, E. T. (2010). Network-based statistic: identifying differences in brain networks. *Neuroimage, 53*(4), 1197-1207. doi:10.1016/j.neuroimage.2010.06.041
